# Dietary Interventions for the Prevention of Type 2 Diabetes in High-Risk Groups: Current State of Evidence and Future Research Needs

**DOI:** 10.3390/nu10091245

**Published:** 2018-09-06

**Authors:** Nicola D. Guess

**Affiliations:** Department of Nutritional Sciences, King’s College London, 150 Stamford Street, Room 4.13, London SE1 9NH, UK; Nicola.Guess@kcl.ac.uk; Tel.: +44-(0)20-7848-4356

**Keywords:** prediabetes, impaired fasting glucose, impaired glucose tolerance, weight loss, fibre protein, Mediterranean, low-carbohydrate, very low energy diets

## Abstract

A series of large-scale randomised controlled trials have demonstrated the effectiveness of lifestyle change in preventing type 2 diabetes in people with impaired glucose tolerance. Participants in these trials consumed a low-fat diet, lost a moderate amount of weight and/or increased their physical activity. Weight loss appears to be the primary driver of type 2 diabetes risk reduction, with individual dietary components playing a minor role. The effect of weight loss via other dietary approaches, such as low-carbohydrate diets, a Mediterranean dietary pattern, intermittent fasting or very-low-energy diets, on the incidence of type 2 diabetes has not been tested. These diets—as described here—could be equally, if not more effective in preventing type 2 diabetes than the tested low-fat diet, and if so, would increase choice for patients. There is also a need to understand the effect of foods and diets on beta-cell function, as the available evidence suggests moderate weight loss, as achieved in the diabetes prevention trials, improves insulin sensitivity but not beta-cell function. Finally, prediabetes is an umbrella term for different prediabetic states, each with distinct underlying pathophysiology. The limited data available question whether moderate weight loss is effective at preventing type 2 diabetes in each of the prediabetes subtypes.

## 1. Introduction

### The Current Paradigm for Type 2 Diabetes Prevention

Current guidelines for the prevention of type 2 diabetes (T2D) in people at high risk are based around achieving moderate weight loss (3–7% weight loss) via dietary change and increasing physical activity [1]. In each of the major diabetes prevention trials (Table 1), dietary advice (for those categorised as overweight) was to lower fat intake to achieve a modest calorie deficit, and to increase physical activity [2,3,4,5,6]. Other dietary changes in some trials included increasing fibre and limiting intake of saturated fat and added sugar [2,6].

These interventions (Table 1) were able to prevent up to two thirds of cases of T2D over the course of the trials, which were 3–6 years in length, with sustained risk reduction compared to the control groups for up to 20 years after the end of the trials [7]. However, within 15 years of the end of the trial, the majority of participants still developed T2D [8]. There are likely several reasons for this including insufficient weight loss and weight regain, a lack of effect of the intervention on beta-cell function, and differences in response depending on the subtype of prediabetes. Given the growing prevalence of prediabetes and T2D worldwide, addressing these issues will be important in helping prevent T2D in more people over the long term. This article will review the evidence to date and make recommendations for future research.

## 2. Interventions to Promote Weight Loss and Weight Loss Maintenance

Overall, these trials and supporting data show that weight loss is the primary driver of T2D risk reduction in people who are overweight [9]. In the US DPP, there was a 96% reduction in risk comparing the 90th against the 10th percentile of weight loss, [9] with each kg of weight loss associated with a 16% reduction in risk [9]. Individual dietary components of these interventions, such as increasing fibre and reducing saturated fat, to date have shown comparatively minimal effect in overweight people compared to simply losing weight [9,10,11], but might play a bigger role in risk reduction for relatively leaner individuals [4,6].

**Figure 1:**
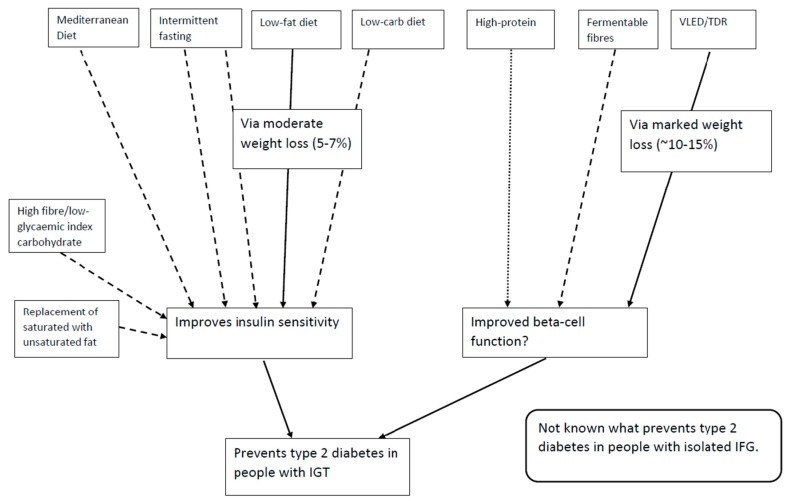
Current dietary strategies shown to improve (full line) or some evidence of improvement (dashed line) and potential for improvement (dotted line) for the two primary pathophysiological defects in the development of type 2 diabetes. IFG: impaired fasting glucose; IGT: impaired glucose tolerance; TDR: total diet replacement; VLED: very low energy diet (<800 kcal/day).

If weight loss is the primary driver of T2D risk reduction, it is possible that other dietary interventions which can lead to >5% weight loss will be as effective as the current guidelines in preventing T2D (Figure 1). Furthermore, long-term weight loss maintenance is more likely to be achieved with a diet that a person enjoys and can stick to. Offering a choice of dietary approach to the patient is therefore important.

### 2.1. Low-Carbohydrate Diets

Data from meta-analyses show that low-carbohydrate diets (the definition can vary but typically under 30% energy from carbohydrate) are at least as good as low-fat diets (<30% of total energy intake from fat) at promoting weight loss [12,13]. Importantly, drop-outs from all weight loss trials are in the order of 35–50% [13,14] and the key factor in whether a participant finishes a trial is whether they like and are able to stick to the lifestyle change. 

There are other aspects of low-carbohydrate diets which could theoretically help prevent the development of T2D in addition to weight loss. Glucotoxicity is defined as the physiological and eventually irreversible β-cell damage caused by chronic exposure to supraphysiological glucose concentrations [15]. Human islets which are experimentally exposed to high concentrations of glucose (11 mmol/L) show reduced insulin biosynthesis, reduced insulin secretion in response to elevated glucose concentrations, and increased rates of insulin release in the presence of low glucose, all of which are characteristics of insulin secretion in vivo in the prediabetic state [16]. Some of these effects can be reversed, depending on the duration of exposure [17]. It is important to note that in these experiments the exposure to hyperglycaemia was continuous and may not reflect daily in vivo glucose profiles. Nonetheless, in mildly prediabetic subjects, blood glucose concentrations may remain over 7.8 mmol/L over an extended post-prandial period [18,19]. It has also been observed that typical consumption of three meals per day (notwithstanding additional snacking or carbohydrate-containing drinks) means that people can spend more than half the day in a post-prandial or post-absorptive state [20]. Marked carbohydrate restriction (~8% of total energy from carbohydrates) is able to rapidly lower post-prandial glucose concentrations in people without T2D [21]. Therefore, although it remains to be tested in prospective trials, dietary interventions which lower post-prandial glucose in people with impaired glucose tolerance (IGT) may be particularly effective at preventing T2D via protecting beta-cell function. The degree of carbohydrate restriction which can meaningfully lower post-prandial glucose concentrations remains to be determined. 

Low-carbohydrate diets could also theoretically protect the beta-cell by reducing insulin demand. It has been proposed that beta-cell exhaustion occurs due to the constant demands of secretion induced by frequent and prolonged episodes of hyperglycaemia [16]. In human islets exposed to continuous hyperglycaemia the use of diazoxide to inhibit insulin secretion helps to prevent hyperglycaemia-induced damage to the islets, and preserves their capacity to synthesise and release insulin [22]. On the other hand, the short-term human data that currently exists suggests that a high-fat diet could impair beta-cell function [23], with in vitro data suggesting saturated fat may be particularly deleterious in this regard [24]. Given the importance of beta-cell function to T2D prevention, this should be urgently studied, and long-term follow-up data is needed.

Ectopic fat is more strongly associated with T2D than BMI, and might play a role in the development or exacerbation of insulin resistance and beta-cell dysfunction [25]. Despite claims that low-carbohydrate diets might help lower ectopic fat deposition, the evidence is currently unclear. Marked restriction of carbohydrates to under 30 g/day appears to lower intrahepatic triglyceride in the absence of weight loss, but it is not clear whether this is due to the high protein content of the interventions [26,27], with the type of fat [28] likely playing an additional role. 

### 2.2. Mediterranean Dietary Pattern

Mediterranean diets have also been shown to help weight loss [29], though again are not superior to any other approach. However, the Mediterranean diet may also help prevent T2D independent of weight loss. 

A pre-planned secondary analysis of the PREDIMED trial showed that a Mediterranean-style diet supplemented with nuts or extra virgin olive oil helps prevent T2D compared to the control diet [30]. This result was striking as the risk reduction occurred in the absence of weight loss. As noted previously, there were very few differences between the control and intervention diets, and any effects appear to be due to the addition of 30 g of nuts or extra virgin olive oil as opposed to regular olive oil [31]. Due to discrepancies in the randomisation procedure, the primary findings of PREDIMED were withdrawn and rewritten, though the outcomes remained materially unchanged. The same appears to be true of the reduction in T2D incidence [32]. There is also limited understanding of the mechanisms of a Mediterranean-style dietary pattern on T2D prevention and these are likely multi-component. Putative mechanisms include improvements in insulin sensitivity via a reduction in inflammation [33], and beneficial effects of fatty acids [34] and phenolic compounds [35] on the beta-cell. A Mediterranean-style diet may also help lower liver fat [36].

Given the proposed beneficial properties of a Mediterranean diet on T2D independent of calorie restriction, combining a weight-reducing diet with a Mediterranean-style diet would be a useful approach. 

### 2.3. Intermittent Fasting 

Intermittent fasting (IF) describes diets which limit calorie intake on certain days (varying in number and whether consecutive or separate), or at certain times [37] (Table 2). Due to the variation in protocols used, interpreting the evidence for these is challenging. Overall, such trials result in the loss of 2.5–9.9% body weight, comparable to weight loss from continuous energy restriction [37]. Drop-out rates are also comparable to other weight loss trials, reaching 40%. The literature on time–restricted feeding (TRF) varies by the time period allocated for energy consumption, and the majority were studies on Ramadan fasting or trials where weight loss was not an aim. Unsurprisingly, these trials show little or only modest changes in weight [38].

There is also interest in proposed specific benefits of IF, particularly on insulin sensitivity. In general, the effect of alternate-day or period fasting on insulin sensitivity is unclear, with some trials showing benefit and others an adverse effect [37]. The majority of data on TRF comes from animal studies, and human studies have largely lacked the robust methodology required to study insulin sensitivity under controlled conditions [38]. However, a recent small but well-controlled crossover trial [39] found that restricting energy intake from 6:00 a.m. to 2:00 p.m. for five weeks in obese males with prediabetes improved insulin sensitivity and increased fat oxidation compared to usual energy intake. This study only included eight people so larger studies involving both genders are required. Nevertheless, given that weight loss and its maintenance are the cornerstones of T2D prevention, IF/TRF diets increase the range of options available to people to achieve weight loss. Any additional physiological benefits remain to be confirmed.

### 2.4. Very-Low-Energy-Diets 

A very low-energy diet (VLED) is defined as a diet with <800 kcal per day. VLEDs consistently produce greater weight loss than other diets [40,41], and contrary to conventional wisdom, rapid weight loss does not increase the likelihood of weight regain [40,41,42]. Very-low-energy diets are associated with better weight-loss maintenance than moderate energy-restricted diets for up to five years of follow-up [42], and greater weight loss [9] and greater weight-loss maintenance [43] are the key drivers of T2D risk reduction.

In addition, a series of physiological trials of varying length have shown that VLEDs can achieve normoglycaemia in people with established T2D via improvements in hepatic and peripheral insulin sensitivity, and via restoration of the first-phase insulin response [44,45,46,47,48,49,50]. Both the amount of weight loss and the rate of weight loss (caloric restriction per se) appear to be independent drivers of the glucose-lowering effect: Better glucose control and insulin sensitivity are observed in people losing 12% of weight on 400 kcal/day compared with 1000 kcal/day [49]. Seven days of 400 kcal/day leads to negligible weight loss, but restores beta-cell function in people with T2D [45]. This confirms findings from bariatric surgery that improvements in glycaemic control occur before any significant weight loss. People with prediabetes already have hepatic and peripheral insulin resistance and impaired beta-cell function [51], and would likely benefit in the same way from these types of interventions [52]. 

The Finnish Diabetes Prevention Study included a VLED as part of the lifestyle intervention [53], but this was only used if participants had not met their weight target. Therefore, a VLED-type approach has not been tested in the prevention of T2D. The cost of delivering resource-intensive one-to-one support in a long-term VLED intervention, such as the recent type 2 diabetes remission trial DiRECT [54], might be prohibitive currently. The national diabetes prevention programme in the UK—the NHS diabetes prevention program [55]—is delivered in groups and with less frequent support, and is therefore less resource-intensive. 

## 3. Interventions to Improve Beta-Cell Function

The two primary defects causing the development of T2D are insulin resistance and beta-cell dysfunction [56] (Figure 1). Moderate weight loss via a low-fat diet (<30% total energy from fat) and moderate physical activity improves insulin sensitivity [57,58,59], which probably helps protect the beta-cell over time via limiting compensatory hyperinsulinaemia [60]. However, post-hoc data from the Finnish and US diabetes prevention studies showed no independent effect of the intervention on the absolute insulin secretory response once changes in insulin sensitivity were taken into account [57,59]. This is important because the seminal event in the conversion from prediabetes to T2D is beta-cell failure [61]. 

The qualitative aspects of beta-cell function in both the fasting and post-prandial state are not fully understood. However, the physiological importance of the pulsatile and first-phase insulin response is clear. 

### 3.1. Pulsatile Insulin Secretion 

It is well established that insulin is secreted by the beta-cell in a pulsatile pattern in the fasting and post-prandial states in approximately 5-min cycles [62]. The pulsatile pattern may help to prevent de-sensitisation of insulin receptors from continuous exposure to insulin. Indeed, insulin infused in a constant versus pulsatile pattern leads to increased endogenous glucose production [63]. The amplitude and frequency of insulin pulses are lost in the prediabetic state [62], and in minimally glucose intolerant relatives of people with T2D [64].

### 3.2. First-Phase Insulin Response 

Following a rapid increase in blood glucose, the beta-cells respond with an immediate, pronounced release of insulin (first phase) which serves to rapidly suppress hepatic glucose output [65,66], followed by a second phase which promotes glucose uptake until normalisation of glucose concentrations is achieved. This biphasic insulin secretion can be clearly observed following intravenous infusion of glucose, but is less well defined following an oral glucose load [65]. 

The physiological importance of the first-phase insulin response is demonstrated by studies in which its experimental suppression results in impaired suppression of hepatic glucose output [67], and higher maximal glucose concentrations [68]. When combined with insulin resistance, these defects lead to prolonged hyperglycaemia which may last for several hours [68]. Conversely, experimental restoration of the absent early insulin response normalises glucose tolerance without increasing the overall insulin demand [68]. Therefore, an impaired first-phase insulin response could (1) place more demand on the beta-cell to control postprandial glucose concentrations (via the second phase); and (2) contribute to a toxic metabolic and hormonal milieu which exacerbates the underlying pathophysiology. Preserving (or restoring) the first-phase insulin response should therefore be a focus of T2D prevention [69]. 

Precise measurement of the first-phase or acute insulin response requires a hyperglycemic clamp, a frequently sampled intravenous glucose tolerance test, C-peptide deconvolution or the use of tracers [70], which to date have been used infrequently. The following section highlights the current evidence base for dietary interventions on beta-cell function. 

### 3.3. Effect of Diet on Beta-Cell Function

The effect of dietary interventions in insulin pulsatility is unclear as there is a near absence of interventional studies which have measured this [71]. There is a limited but growing body of research on the effects of diets and nutrients on the first-phase insulin response. 

As described above, marked energy restriction and/or weight loss can restore the first-phase insulin response in people with established T2D [45,46,50], but moderate weight loss or physical activity does not appear to [57,58,72,73,74]. The mechanism is currently unclear, but might be due to reducing ectopic fat [50], reducing glucotoxitciy [15,16] and allowing the beta-cell to rest [75].

There is limited data suggesting that dietary fibre may improve beta-cell function [76,77,78,79] (Figure 1), but this has not been studied extensively. The effect may be via increasing glucagon-like peptide-1 (GLP-1) [79] or by a direct effect of short-chain fatty acids (produced by colonic fermentation of fibre) on the beta-cell [80]. The type of fibre or combinations of fibres which are most effective is not clear currently. 

Protein may potentiate insulin secretion via the incretin hormones gastric inhibitory polypeptide (GIP) and glucagon-like peptide-1 (GLP-1) [81]. In addition, amino-acid-specific insulin secretion may also play a role. Amino acids act alone or synergistically with glucose to potentiate the release of insulin [82], and while amino-acid-stimulated insulin secretion (AAIS) shares some pathways with glucose-stimulated insulin secretion (GSIS), some pathways are distinct [83]. There are some acute and chronic studies which have estimated insulin secretion via an oral glucose tolerance test which suggest that protein foods may help the secretion of insulin post-prandially [84,85], but further studies are required to confirm this. A note of caution is required given findings that a higher-protein diet may ameliorate weight-loss-induced improvements in insulin sensitivity compared to a lower-protein diet [86], and the clinical significance of this long-term is unknown.

Eight weeks of a high-carbohydrate (CHO) (55% CHO; 27% fat) diet in overweight men and women with prediabetes improved the beta-cell response to glucose compared to a lower-CHO, higher-fat diet [87]. (43% CHO; 39% fat). However, two weeks of a high-fat, moderate-carbohydrate diet (43% fat and 40% carbohydrate) had no effect on the acute insulin response in healthy normoglycaemic males compared to a low-fat, high-carbohydrate diet (25% fat and 56% carbohydrate) [88]. The fibre content of the meals in these studies differed, which may have played a role in modulation of beta-cell function.

There is therefore currently not sufficient evidence to make nutrient-based recommendations to improve beta-cell function (Figure 1). Given the importance of beta-cell function to the pathogenesis of T2D, this area of research should be prioritised.

## 4. Interventions Which Target the Prediabetic Subtype

Prediabetes is an umbrella term for at least two conditions: impaired fasting glucose (IFG) and impaired glucose tolerance (IGT). IFG is defined by the WHO as a fasting plasma glucose (FPG) of >6.1 mmol/L, while IGT is a glucose concentration two hours after a 75 g oral glucose tolerance test of 7.8–11.0 mmol/L [89]. A person can have elevated FPG with normal 2-h glucose concentration (isolated-IFG), or a normal FPG with elevated two-hour plasma glucose (isolated IGT) or with both FPG and two-hour glucose elevated (IFG/IGT).

The current guidelines for T2D prevention are based on the results of lifestyle interventions which were all carried out in populations with IGT (with or without IFG) [2,3,4,5,6] (Figure 1). A T2D prevention study in Japan [90] also included a sub-group with isolated IFG (I-IFG) and found that the intervention did not lower the risk of T2D in this group. The adjusted hazard ratio (HR) was 1.17 (95% confidence interval (CI), CI 0.50–2.74) in the I-IFG group compared to 0.41 (0.24–0.69) in the IFG/IGT group. However, the incidence of T2D in the I-IFG group was six times lower than the incidence in the IFG/IGT group, suggesting that this group was at relatively low baseline risk [90].

The D-Clip study also included people from all subtypes of prediabetes and also found non-significant risk reduction in the I-IFG group (12%) compared to I-IGT (31%) and IFG/IGT (36%) groups [91]. Moreover, the study design included a plan for the introduction of metformin in participants at highest risk of conversion to diabetes at ≥4 months of follow-up. The proportion of people with I-IFG requiring metformin was higher (77%) than the I-IGT group (51%) [91]. This might explain the absence of protective effect.

A peer-support T2D prevention study in India [92] also found no risk reduction in the I-IFG group and a post-hoc analysis of the Finnish Diabetes Prevention Study found differences in the effect of the intervention on fasting versus two-hour glucose concentrations, which also differed between people with IFG/IGT and people with I-IGT [93]. It is important to note that none of these studies had an a priori hypothesis to test whether the effect of the intervention was different between prediabetes subtypes, and none were powered to do so. However, the evidence is consistent in suggesting that the prediabetes subtype could mediate the effectiveness of lifestyle change to prevent T2D. The prevalence of I-IFG may reach 10% of the population [94], and approximately one fifth of people develop T2D via elevated FPG with a normal two-hour glucose concentration [95]. Understanding the effect of lifestyle in preventing T2D via I-IFG is therefore an important public health question (Figure 1).

The distinct differences in underlying pathophysiology may help to explain why lifestyle change could affect progression to T2D to different degrees. IFG is characterised by marked hepatic insulin resistance, elevated hepatic glucose output, but normal muscle insulin sensitivity [96]. Conversely, in IGT there may be mild hepatic insulin resistance and marked muscle insulin resistance. There are also differences in insulin secretion—in IGT there are defects in the first and second-phase insulin responses, while in IFG the first-phase response is defective, but the second-phase remains intact [51].

Research into the effect of diet on the underlying pathophysiology of T2D will provide insight into the larger question of preventing T2D and also whether it is possible to optimise T2D prevention by targeting diet to the underlying pathophysiology.

## 5. Summary

The totality of evidence from diabetes prevention studies worldwide shows the importance of weight loss in the prevention of T2D. The dietary intervention used in these studies has been tested in multiple populations, ethnicities and settings, and represents the strongest evidence base currently. On the other hand, it is also true that the dietary intervention used in these interventions has not been compared to other diets. This work should be undertaken. This will not only increase choice for patients, and therefore potentially adherence, but may also reveal approaches which may be more effective than the current standard low-fat diet. It is also necessary to understand how diet affects the underlying pathophysiology. By identifying the mechanism of the effect of foods and nutrients on beta-cell function and tissue-specific insulin sensitivity, we could then design and test interventions which target the underlying physiological defects. This may be particularly relevant to prevention of T2D in people with isolated-impaired fasting glucose, in whom the effectiveness of current diabetes prevention programs has not been demonstrated.

## Figures and Tables

**Table 1:** Brief description of the dietary and lifestyle changes included in the major type 2 diabetes prevention trials.

Name of Study	Intervention
The Finnish Diabetes Prevention Study (FDPS) (2)	Aim for 5% weight loss, fat <30% kcal intake, saturated fat <10% kcal intake, fibre >15 g per 1000 kcal, PA: 30 min/day.
U.S. Diabetes Prevention Program (DPP) (3)	Aim for 7% weight loss, fat <25% kcal intake, PA: 150 min/week.
The Da-Qing Impaired Glucose Tolerance (IGT) and Diabetes Study (4)	High-carbohydrate and low-fat diet, ↑ PA by 12 units/day. Aim for 23 kg/m^2^ if BMI > 25 kg/m^2^.
Japanese Diabetes Prevention Trial (5)	Reduce BMI to 22 kg/m^2^. Dietary advice individualised, ↓ fat intake (<50 g/day), portion size, alcohol intake, ↓ eating out. PA: 30–40 min/day.
Indian Diabetes Prevention Study (6)	Avoid simple sugars and refined carbohydrate, fat <20 g/day, ↑ fibre. PA: 30 min/day.

BMI: body mass index; PA: physical activity; ↑: increased; ↓: decreased.

**Table 2:** Summary and brief description of types of intermittent fasting (IF). Adapted from [37] and used with permission.

Type of Intermittent Fasting	Description
Alternate day fasting	Alternating feast (*ad libitum* intake) and fast days (≤25% of energy needs)
Time-restricted fasting	Eating only during certain time periods (i.e., 8 h), then fasting for remaining hours of the day
Periodic fasting	Fasting for up to 24 h once or twice a week with *ad libitum* intake on the remaining days
